# Risk control of host cell proteins in one therapeutic antibody produced by concentrated fed‐batch (CFB) mode

**DOI:** 10.1002/elsc.202200060

**Published:** 2023-02-07

**Authors:** Yiling Lu, Jun Lin, Tianze Bian, Jin Chen, Dan Liu, Mingjun Ma, Zhen Gao, Jiemin Chen, Dianwen Ju, Xing Wang

**Affiliations:** ^1^ Department of Biological Medicines & Shanghai Engineering Research Center of Immunotherapeutics Fudan University School of Pharmacy Shanghai China; ^2^ Department of Analytical Science Formulation & Quality Control, Genor Biopharma Co., Ltd. Shanghai China; ^3^ Array Bridge Inc. St. Louis Missouri USA

**Keywords:** concentrated fed‐batch (CFB), ELISA, host cell proteins (HCPs), mass spectrum (MS), risk control

## Abstract

Multiple control strategies, including a downstream purification process with well‐controlled parameters and a comprehensive release or characterization for intermediates or drug substances, were implemented to mitigate the potential risk of host cell proteins (HCPs) in one concentrated fed‐batch (CFB) mode manufactured product. A host cell process specific enzyme‐linked immunosorbent assay (ELISA) method was developed for the quantitation of HCPs. The method was fully validated and showed good performance including high antibody coverage. This was confirmed by 2D Gel‐Western Blot analysis. Furthermore, a LC‐MS/MS method with non‐denaturing digestion and a long gradient chromatographic separation coupled with data dependent acquisition (DDA) on a Thermo/QE‐HF‐X mass spectrometer was developed as an orthogonal method to help identify the specific types of HCPs in this CFB product. Because of the high sensitivity, selectivity and adaptability of the new developed LC‐MS/MS method, significantly more species of HCP contaminants were able to be identified. Even though high levels of HCPs were observed in the harvest bulk of this CFB product, the development of multiple processes and analytical control strategies may greatly mitigate potential risks and reduce HCPs contaminants to a very low level. No high‐risk HCP was identified and the total amount of HCPs was very low in the CFB final product.

**Abbreviations**
CFB1: concentrated fed‐batch 1
DDA3: data dependent acquisition 3
DTT6: Dithiothreitol 6
ELISA5: enzyme‐linked immunosorbent assay 5
FB7: fed‐batch 7
HCPs2: host cell proteins 2
MS4: mass spectrum 4

## 1 INTRODUCTION

Most therapeutic antibodies produced by inserting the target gene into a plasmid system and having a mammalian host cell express and translate the gene [1]. However, a large number of host cell proteins (HCPs) are co‐expressed and contaminate therapeutic antibody drugs during cell culturing processes [2, 3]. There have been numerous reports covering the risk of HCPs which include immunogenicity or reducing drug efficacy, etc. [4, 5, 6, 7, 8]. Therefore, the HCP levels in the final products should be reduced as much as possible and must be monitored and controlled to acceptable levels to mitigate potential risks [9, 10]. Combinations of several different purification steps could provide the most efficient removal of HCPs. Multistep chromatographic and filtration processes can eliminate thousands of host proteins from the target protein based on differences of charge, size, hydrophobicity, and other properties [11, 12, 13, 14]. Typical purification processes usually include protein‐A affinity chromatography (PA), viral inactivation (VI), cation exchange chromatography (CEX), anion exchange chromatography (AEX), depth filtration (DF), and ultra‐filtration (UF) [15, 16, 17].

Though large amounts of HCPs were removed by multistep purification strategies, there were still residual HCPs co‐purified with the target protein that remained in our target products. Monitoring these trace contaminants in the final drugs was challenging. Multiple assays with high sensitivity and specificity need to be implemented. Due to its high specificity, accuracy, precision, and automation, enzyme‐linked immunosorbent assay (ELISA) has been widely used to quantify HCPs in the biopharmaceutical industry [18, 19, 20]. However, ELISA [20, 21, 22], does not fully cover all HCPs from the host cell line and is biased towards higher immunogenic HCPs. The current quantitation platform, the microplate‐based ELISA, can only bind about 1 μg of antibody in each well. Considering the antibodies used are derived from hundreds to thousands of HCPs, the antibody concentration for any specific HCPs is relatively low, therefore, only the relatively abundant HCPs with good immunogenicity was detected. Another challenge is that the current format of ELISA is a sandwich ELISA, which requires that the detected HCP must produce two non‐interfering antibody pair, for many HCPs, this may not be the case. Furthermore, the quantitation of the sandwich ELISA relies on the antibody concentration, that is, the detection is not a direct measurement of the mass of HCPs, instead it is a measurement of HCP immunogenicity and then indirectly deduces the amount of HCPs in the test. A process‐specific HCP ELISA with increased antibody coverage could partially mitigate some of the aforementioned challenges but further improvements in HCP detection technologies are warranted.

As an alternative method to ELISA, LC‐MS/MS combines a high separation liquid phase system with a high‐resolution mass spectrometry system and enables HCP proteins to be separated by LC and identified by mass spectrometers. The analysis and identification of HCPs by the LC‐MS/MS method could sensitively detect HCPs independent of their immunogenicity [23, 24, 25, 26, 27]. However, considering the interference of large amounts of target protein, detecting these trace amounts of HCPs (<1–100 ng HCP per mg drug products) directly by mass spectrometry is still challenging. Therefore, multiple techniques in sample preparation and instrumental analysis have been developed and optimized to decrease the interference of drug products and increase the abundance of HCP proteins. Through optimization of the amount of digestion buffer, concentration of trypsin, LC gradient, and column/data acquisition modes, improvements were achieved by digesting HCPs prior to the target protein as well as separating and identifying more co‐eluted peptides, etc. [27, 28].

**Practical Application**

In this paper, we reported the development of two methods to mitigate the risks of HCP in one concentrated fed‐batch (CFB) mode product. To our knowledge, this was the first paper to discuss risk mitigation strategies for HCP in CFB products using this unique approach. In this report, one host cell and process specific enzyme‐linked immunosorbent assay (ELISA) method were developed with excellent coverage for total HCP determination. Another LC‐MS/MS method with non‐denaturing digestion and a long gradient chromatographic separation coupled with data dependent acquisition (DDA) was developed to identify specific types of HCPs. By monitoring the clearance trends of HCPs from harvest bulk, the risks can be more easily controlled. We believe that this study provides valuable insight on how to manage potential risks in CFB production of biologics and should be valuable for scientists from industries as well as academics working on for this new mode of bioprocess.

Compared to traditional FB, perfusion culture manufacturing technology allows for maximum productivity and cost reduction. Perfusion culturing technology has been quickly adopted in the biopharmaceutical industry. Either the concentrated fed‐batch (CFB) mode [29, 30] or continuous perfusion cell culture mode [31] can be used in the production stage to enhance productivity. However, the higher cell density culturing technology might bring more host cell related impurities such as HCPs and host cell DNAs (HCDs), especially in the CFB mode. It is more challenging to purify this kind of product than others from traditional FB processes. Therefore, the risks of HCPs in the CFB products exist and the strategy to select and use suitable methods to control the HCPs in both identification and quantitation ways are greatly required.

In this paper, a comprehensive characterization strategy involving a host cell, process specific ELISA method, and method with novel non‐denaturing enzyme digestion and long chromatographic LC‐MS/MS technology were designed to characterize low abundance HCPs (<100 ppm) in one CFB‐mode product. Both methods demonstrated good performance including high sensitivity, selectivity, and adaptability. They also demonstrated the decreasing trend of HCPs through all the purification steps. Finally, only trace levels of HCPs of low potential risk were identified in the final product. With a combination of process and analytical control strategies, the risk of HCPs in a CFB product can be greatly mitigated.]

## 2 MATERIALS AND METHODS

### 2.1 Materials

CHO null strains and monoclonal IgG4 were produced in‐house (Genor, ShangHai, China). Bovine trypsin was obtained from Promega (Madison, USA). Dithiothreitol (DTT) was from Bio‐Rad (Canada). Other conventional reagents, like formic acid and Tris‐HCl were purchased from Fisher Scientific (Rockford, IL) and Invitrogen (Grand Island, USA), respectively. The reagent used in this study is RapiGest SF made by Waters (Milford, USA). All the reagents used in our experiments were reagent grade or higher purity.

### 2.2 Methods

#### 2.2.1 Specific HCPs determination ELISA method development and validation

Generally, the null vector was transfected into CHO‐S strain and cultured by the CFB process. Then, all the HCPs from CHO null strains were purified and used to immunize rabbits. Next, the rabbit pAb obtained from animal immunization was purified and tested by an optimized specific ELISA method. After several rounds of experimental optimization, the final conditions of this specific ELISA method were determined as follows: The concentration of coated polyclonal antibody was 5 μg/mL. The range of HCP standards was 1.1∼810 ng/mL. The concentration of biotin‐labeled polyclonal antibody was at 2 μg/mL. The concentration of HRP‐Streptavidin was at 0.05 μg/mL (1:20000). The reaction was conducted at room temperature for 4 min, and the reading was performed at 450 nm after termination with 1 M sulfuric acid.

#### 2.2.2 Sample preparation for CHO null strain

An aliquot containing 2.5 mg of CHO null strains protein solution was mixed with 0.05% RapiGest SF and 50 mM ammonium bicarbonate at 60°C for 15 min. The solution was then reduced with 20 mM DTT and incubated at 60°C for 60 min followed by alkylation with 10 mM IAM for 30 min in the dark. Finally, the denatured and reduced proteins were digested with trypsin (1:40 w/w trypsin: protein) at 37°C overnight, and finally quenched by acidifying with 0.5% FA.

#### 2.2.3 Sample preparation for product intermediates

CFB process intermediates were diluted with Tris‐HCl buffer (50 mM, pH 7.6) to about 3 mg/mL. These diluted solutions were then digested under non‐denaturing conditions with different amounts of trypsin (1: 200∼1: 5000 w/w trypsin: protein) at 37°C for 2 h, reduced with 10 mM DTT and heated at 95°C for 5 min. Precipitation of undigested proteins was achieved by centrifuging at 13,400 rpm for 15 min. The supernatant was then quenched by acidifying with 0.25% FA and dried by spin dryer at 40°C and 1500 rpm. The samples were then sufficiently dissolved by 150 μL of ultrapure water.

#### 2.2.4 LC‐MS/MS method development and analysis

For the LC‐MS/MS method, the columns and gradients were optimized to obtain a good separation for the low abundance HCPs. A 2.1 × 150 mm or a 1.0 × 150 mm CSH130 C18 Acquity UPLC Column (1.7 μm, 130 Å) was used with the total gradient time of 120 min or 160 min and the column temperature was 60°C. The first separation gradient was increased from 1% to 10% mobile phase B (100% acetonitrile with 0.1% FA) over 25 min. This was followed by 20% B over 55 min, 30% B over 80 min and 37% B over 95 min at 300 μL/min. The solution was then washed with 80% B at 300 μL/min for 10 min. By lowering the flow rate from 200 to 150 μL/min during the equilibrium and linear elution processes, the final separation was performed on the 2.1 × 150 mm CSH130 C18 Acquity UPLC Column (1.7 μm, 130 Å). The column equilibrated at mobile phase B (100% acetonitrile with 0.1% FA) for 3 min at 150 μL/min and linearly increased from 1% to 10% B over 40 min. At a flow rate of 150 uL/min, this was followed by an increase to 20% B over 85 min, 30% B over 120 min and 37% B over 135 min. Next, there was a 10 min washing process with 80%B and 20% mobile phase A (100% water with 0.1% FA) at a flow rate of 250 μL/min. All peaks eluting from the chromatography column were analyzed using a Thermo QE‐HFX mass spectrometer with an ESI source operating in a positive, high‐resolution model (Full MS resolution of 120,000; dd‐MS2 resolution of 30,000). Other parameters included a scan range of 200 to 2000 m/z, capillary temperature of 320°C, spray voltage of 3.8 kV, and aux gas heater temperature of 350°C. The flow rate of sheath gas and aux gas were set at 40 and 10 arb, respectively. The parameters of full MS and dd‐MS2 in one cycle of DDA mode were performed for HCP identification and listed as follows: Full MS scans were operated in profile mode with microscans of 1 and a full scan AGC target of 3E6. The first through tenth most abundant ions (Top *N*  =  10) were detected at a minimum intensity threshold of 1E4 and NCE of 27. The dd‐MS2 scans were collected in centroid mode with the maximum IT of 100 ms, AGC target of 1E5, and dynamic exclusion time of 15 ms. Proteome Discoverer 2.4 software purchased from Thermo Fisher Scientific was used in database searches for HCP identification.]

## 3 RESULTS AND DISCUSSION

### 3.1 Harvest fluid produced by CFB mode showed higher HCP levels than FB mode

**FIGURE 1:**
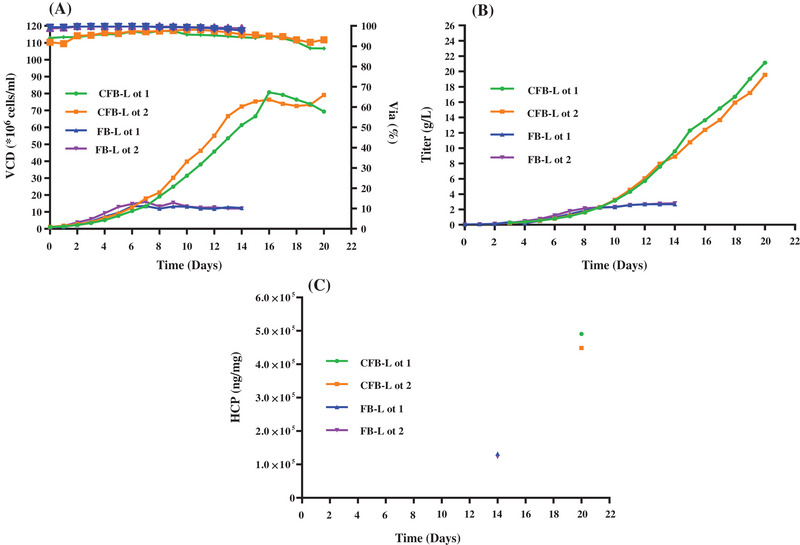
Comparison of concentrated fed‐batch (CFB) mode process and fed‐batch (FB) mode process (A) Comparison of viable cell density and viability curves for 2 CFB lots and 2 FB lots; (B) comparison of titer curves for 2 CFB lots and 2 FB lots; (C) comparison of host cell proteins (HCPs) amounts in harvest bulks for 2 CFB lots and 2 FB lots.

**FIGURE 2:**
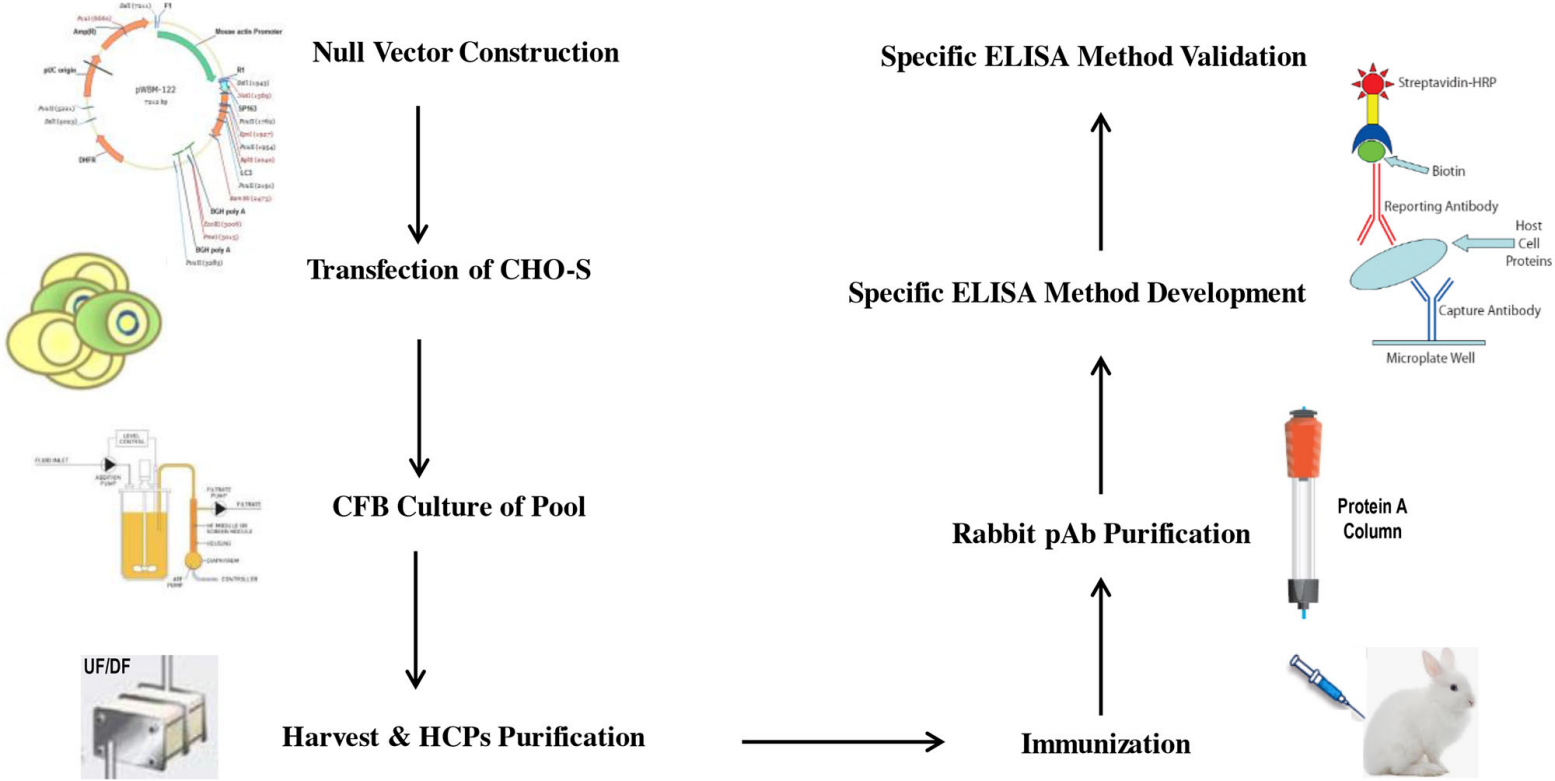
Workflow of in‐house host cell proteins (HCPs) determination kit method development.

Different from the traditional FB culture process, the new CFB culture process achieved much higher cell densities and titers by continuously replenishing fresh medium while simultaneously intercepting cells and products in the reactor through a small aperture hollow fiber column. The CFB process was continuous with cell and antibody retention by 50 KD hollow fiber module with the cell specific perfusion rate (CSPR) 10–50 pl/cell/day. The feed was continuous with the media self‐prepared. The cell culture was harvested and centrifuged at 6500 g for 25 min and the supernatant was then collected and deep filtered with Millistak+ Pod D0HC (Merck KGaA) and Millistak+ Pod X0HC (Merck KGaA) with both total areas of 2.2 m^2^ at the rate of 0∼200 LMH (liter/m^2^/hour). This high‐density culture process would inevitably produce more HCP contaminants than the FB process. As shown in Figures 1 and 2, the CFB process yielded a cell density of about 7 × 10^7^ cells/mL, nearly 7‐fold higher than that of the FB processes while both processes maintained high cell viability of ≥90%. Accordingly, the titers of the CFB process reached about 20 mg/mL, about 8‐fold higher than that of a traditional FB process. However, the total amounts of HCPs in harvest fluid of the CFB process were about 600,000 ppm, nearly 6‐fold higher than that of FB process, which indicated the potential high risk of HCPs in CFB mode processes and the increased challenges of removal and control of HCPs in the final product. The cell free culture fluid was stored at ≤−60°C before ELISA/MS experiments.

### 3.2 Specific HCPs determination ELISA method showed excellent performance including high recovery and other acceptable validation properties

**FIGURE 3:**
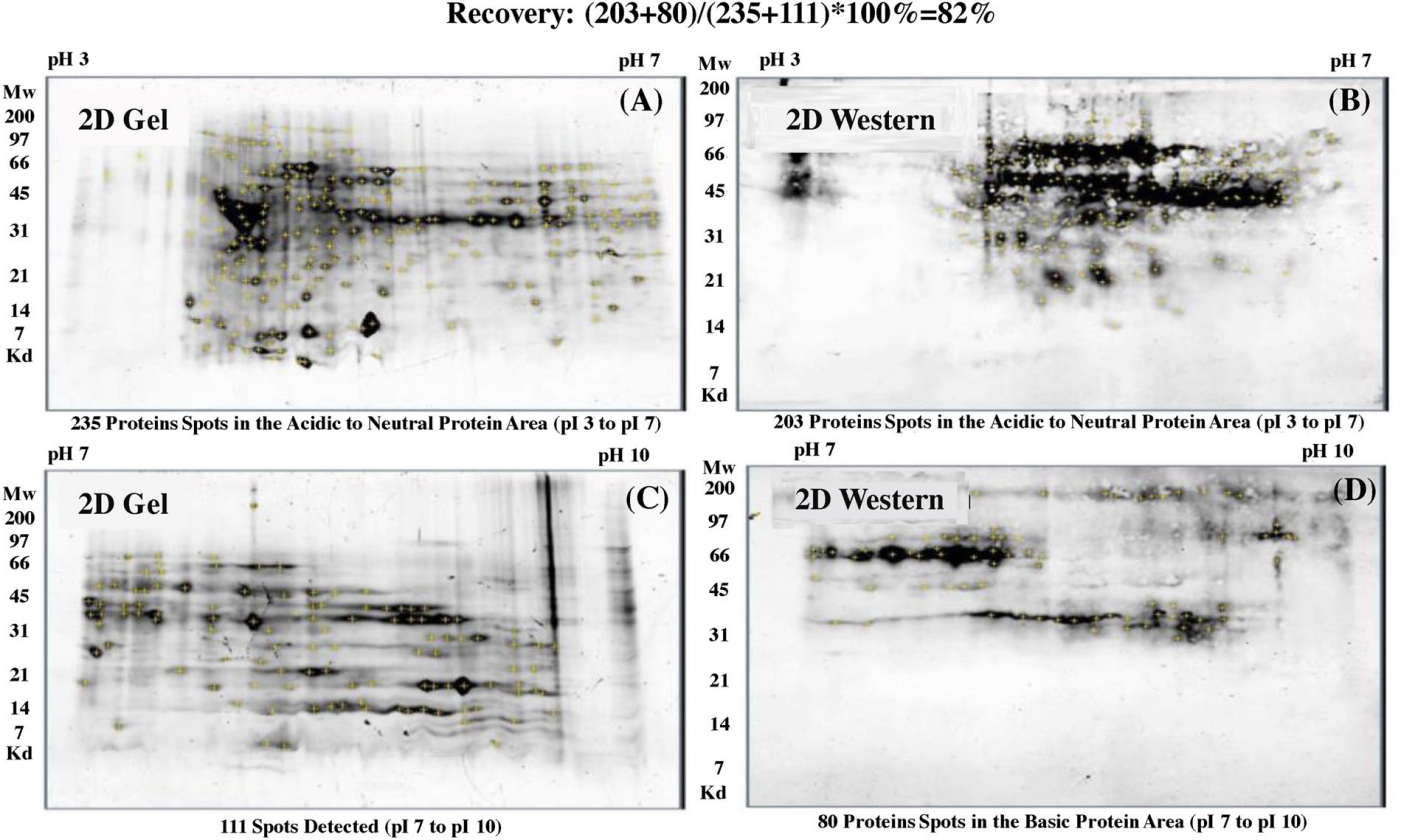
Coverage determination by 2D‐Gel‐Western Blot for in‐house host cell protein (HCP) kits (A) 2D Gel analysis for detecting acidic to neutral pH CHO HCPs stained with Sypro Ruby, cut and analyzed; (B) Western Blot analysis of anti‐CHO HCP antibody covering acidic to neutral pH HCPs, cut and analyzed; (C) 2D Gel analysis for detecting neutral to basic pH CHO HCPs stained with Sypro Ruby, cut and analyzed; (D) Western Blot analysis of anti‐CHO HCP antibody covering neutral to basic pH HCPs, cut and analyzed.

Considering the high risk of HCPs in the CFB process, the capabilities of purification steps to clear HCPs are very critical. Furthermore, applying multiple analytical control strategies would also mitigate the risk of HCPs. More species of HCPs would be certainly detected since its higher concentration for CFB compared to FB. Furthermore, due to more cultivation days with higher cell densities, CFB would also possibly produce more species of HCP. Therefore, detection or identification of most HCPs produced by this non‐traditional process are potential challenges. The proteins harvested from null vector cell trains at CFB mode were selected for HCP analysis with commercial generic HCPs. However, the antibody coverage detected by a 2D‐Western Blot was lower than 40%, which would increase the possibility to fail to detect some high‐risk HCPs. Thus, an in‐house host cell specific and process specific HCP determination kit is greatly required. The in‐house kit method development flow is described in Figure 2. First, null vector was constructed and transferred into CHO‐S cell. The engineered cell pool was cultured at a CFB mode and the cell culture was harvested and centrifuged at 6500 g for 25 min and the cell supernatant was collected and then buffer exchanged into PBS through UF/DF at cutoff of 10 KD (Millipore), which would retain most of the HCPs. Though some of the HCPs have molecular weight less than 10 KD in SDS‐PAGE, they are actually in the form of multimers or complexes in native. Almost no protein was detected in the membrane permeate, which indicated the HCP loss in the UF/DF might be acceptable. The purified HCPs were injected into the rabbits and then serum of the rabbits after immunizations were collected. Six rabbits were immunized with the staring dose of 4 mg of HCP combined with 1 mL of complete Freund's adjuvant. For the subsequent multiple intramuscular injections, 4 mg of HCP combined with 1 mL of incomplete Freund's adjuvant were dosed. The total immune period was 98 days. The rabbits’ serum sample was diluted 1:1 with PBS and loaded onto the Cytiva's MabSelect affinity column with loading capacity of 17.5 mg proteins/mL resin. The column was washed with 100% Buffer A (PBS) for 10 min and then the proteins were eluted with the increasing Buffer B (50 mM Glycine, pH 2.8) gradient from 0% to 100% in the following 40 min. The flow rate was 2 mL/min. The purified polyclonal antibodies were thus acquired. The biotin labeled polyclonal antibodies were used as capturing antibodies and reporting antibodies respectively. A sandwich ELISA method was developed and then fully validated following the ICH Q2 guideline. All the results (listed in Table 1) demonstrated that the method has excellent performance including good specificity, linearity, accuracy, repeatability, and robustness. The antibodies prepared from the host cell and process specific HCPs showed higher coverage (77%) than that of the commercial generic kit (37%) toward the HCPs prepared from the null cells, indicating improved suitability of this self‐developed method (Figure 3).

**TABLE 1:** Validation results of specific ELISA method

Item	Acceptance criteria	Result
Coverage	≥60% confirmed by 2D electrophoresis and western blot	77%
Specificity	Recovery: 70%–130%, RSD ≤ 30.0%	Recovery: 100%, RSD: 9.3% (*N* = 9)
Linearity	*R* ^2^ ≥ 0.990 at specific concentrations	Linearity at range of 0.37∼270 ng/mL, *R* ^2^ ≥ 0.999 (*N* = 3)
Range	The range meeting the recovery of 70%–130% and RSD ≤ 25.0%	1.0∼15.0 ng/mg
Accuracy	Recovery 70%–130% and RSD ≤ 25.0% for 5 concentrations of spiked HCPs standard	1) Recovery 103%, RSD 4.9% with adding conc. of 1.0 ng/mg (*N* = 6)
		2) Recovery 120%, RSD 11.0% with adding conc. of 2.0 ng/mg (*N* = 6)
		3) Recovery 120%, RSD 9.7% with adding conc. of 5.0 ng/mg (*N* = 6)
		4) Recovery 119%, RSD 2.4% with adding conc. of 10.0 ng/mg (*N* = 6)
		5) Recovery 118%, RSD 14.1% with adding conc. of 15.0 ng/mg (*N* = 6)
Repeatability	RSD of 6 tests ≤25.0% for DS spiked with 1.0 ng/mg HCPs standard	RSD 4.9% (*N* = 6)
LOQ	The lowest spiked HCPs standard conc.to meet accuracy requirements	1.0 ng/mg
Robustness	RSD ≤ 30.0% for different coloring times (3.5, 4.0, and 4.5 min)	RSD 10.7%

Abbreviations: ELISA, enzyme‐linked immunosorbent assay; HCPs, host cell proteins.

### 3.3 LC‐MS/MS method showed improved performance through multiple parameters optimization for the identification of HCPs in CHO null strain and purification intermediates

**FIGURE 4:**
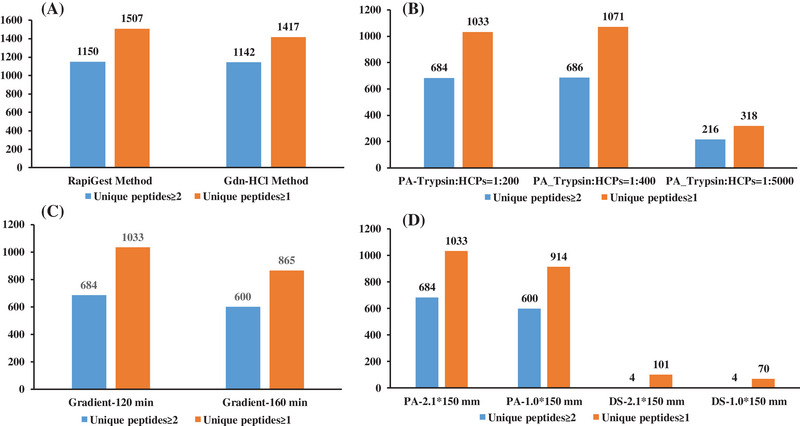
Trypsin amount, columns, and gradients optimization of LC‐MS/MS method for host cell proteins (HCPs) determination in purified intermediates (A) Different denaturing reagent methods including RapiGest SF method and Gdn‐HCl method were compared. B) Different amounts of trypsin (ratios of 1:200, 1:400, and 1:5000 w/w trypsin: protein) were chosen for HCP analysis in protein A affinity chromatography (PA) intermediates. Each sample was run in triplicate. (C) Total 120 min gradients and total 160 min gradients were optimized to improve HCP identification. Prolonging gradients to 160 min did not improve HCP identification. (D) Two columns (2.1 × 150 mm column and 1.0 × 150 mm column) were used to optimize the HCP analysis.

We used denaturing digestion condition for CHO null strain sample analysis and native digestion conditions for purification intermediates. Considering operation convenience for protein denaturing and that we identified more CHO null strain HCPs, RapiGest SF was superior to Gdn‐HCl, as shown in Figure 4A. RapiGest SF was thus selected as the denaturing reagent. For HCPs in the intermediate analysis, ratios of 1:200, 1:400, and 1:5000 (w/w trypsin: protein) for native digestion conditions were first screened to obtain the maximum amount of HCPs. As shown in Figure 4B, the condition of 1:200 (w/w trypsin: protein) obtained similar HCPs to the condition of 1:400 (w/w trypsin: protein) but better than that of 1:5000 (w/w trypsin: protein). However, to assure complete digestions every time, the condition with more trypsin (1:200) was subsequently used for further HCP detection of purification intermediates. Furthermore, Acquity UPLC CSH130 C18 Columns (1.7 μm, 130 Å) screening was performed. As shown in Figure 4C and D, more HCPs were identified by the 2.1 × 150 mm column in both the Protein A purified intermediate and final drug substance. Thus, this column was selected to be used in further studies. The total gradient time was maintained at 120 min as increasing the gradient time to 160 min did not yield improved separation. After the above optimizations, the acquired traditional LC‐MS/MS method showed comparable results to the nanoLC‐MS/MS coupled with BoxCar acquisition method [32].

### 3.4 HCPs in CHO null strains and purification intermediates and drug substance analyzed by both specific ELISA method and LC‐MS/MS method

**FIGURE 5:**
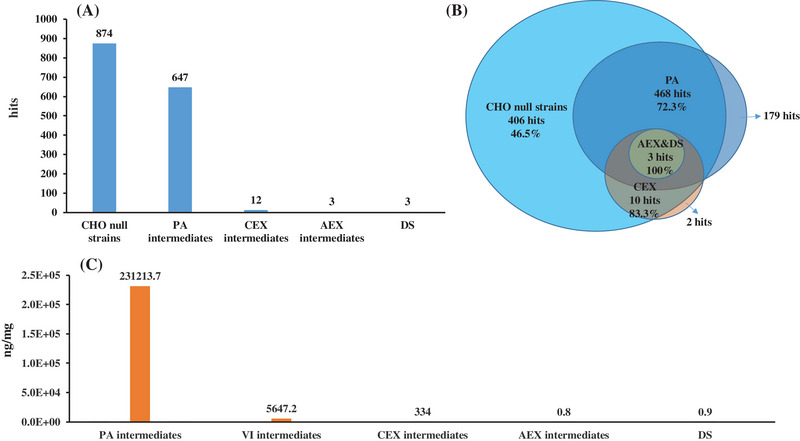
All the host cell proteins (HCPs) from CHO null strains and different purified intermediates were identified by LC‐MS/MS and ELISA (A) The HCP species in different purified intermediates (CHO null strains vs. PA vs. CEX vs. AEX vs. DS) were detected by LC‐MS/MS method. (B) Venn diagram of the total HCP species identified by LC‐MS/MS in CHO null strains and different purified intermediates also illustrate the satisfactory removal rate of HCPs by different purified steps. (C) The total amount of HCP in different purified intermediates was detected by ELISA method. AEX, anion exchange chromatography; CEX, cation exchange chromatography; PA, protein‐A affinity chromatography.

HCPs of CHO null strains and all purification intermediates from the same manufacturing lot were analyzed by both the specific ELISA method and optimized LC‐MS/MS method. A condensed histogram and pie chart were constructed, demonstrating a good removal rate of HCPs from the CHO null strains and all purification intermediates (Figure 5A). As shown in Figure 5A and B, about 46.5% more HCPs (874 vs. 647) were identified in the CHO null strains than the PA intermediates, and 72.3% HCPs in PA intermediates were also identified in the CHO null strains. These results (CHO null strains vs. PA vs. CEX vs. AEX vs. DS = 874 vs. 647 vs. 12 vs. 3 vs. 3) demonstrated that our purification process has good clearance capabilities for HCPs. Furthermore, as shown in Figure 5C, the results of host and process specific ELISAs showed the same decreasing trend of total amount of HCPs with very little HCPs detected in the final product. The detected HCPs in the final purified intermediates (DS) are listed in Table 2. No high‐risk HCPs were found among these intermediates. Furthermore, we also compared the commercial HCP ELISA assay data, however, the commercial HCP ELISA assay overestimated the HCPs from the bioprocess by about 3 times as compared with the self‐developed assay (data not shown). One of the possible reasons was that in the self‐developed ELISA, the HCP standards was freshly prepared while in the commercial ELISA kit, the HCP standards was prepared in advance for several weeks or many months in the presence of high protein concentration carrier protein which was used to prevent loss of the nanogram level HCPs. During the storage of the extremely low level of HCPs in the solution with carrier protein, the HCPs tend to form complexes thus reducing the detection.

**TABLE 2:** All the HCPs identified in the 2 CFB DS lots and high‐risk HCPs not detected

HCPs	Description	Lot 1	Lot 2
UBE2F	KRT77 OS = Cricetulus griseus OX = 10029 GN = CgPICR_000226 PE = 3 SV = 1	√	√
KRT10	KRT10 OS = Cricetulus griseus OX = 10029 GN = CgPICR_017009 PE = 3 SV = 1	√	√
Phospholipase B‐like	Phospholipase B‐like OS = Cricetulus griseus OX = 10029 GN = CgPICR_012279 PE = 3 SV = 1	√	√
**PLBL2** **^a^**	Phospholipase B‐like OS = Cricetulus griseus OX = 10029 GN = CgPICR_012279 PE = 3 SV = 1	ND	ND
**MCP‐1** ^a^	Monocyte chemoattractant protein‐1 OS = Cricetulus griseus GN = CCL2	ND	ND
**Protein disulfide isomerase** ^a^	Protein disulfide‐isomerase OS = Cricetulus griseus OX = 10029 GN = CgPICR_011940 PE = 3 SV = 1	ND	ND
**Ubiquitin** ^a^	Ubiquitin OS = Cricetulus griseus OX = 10029 GN = I79_017072 PE = 4 SV = 1	ND	ND
**Clusterin** ^a^	Clusterin OS = Cricetulus griseus OX = 10029 GN = I79_012331 PE = 3 SV = 1	ND	ND
**LPLA2** ^a^	Phospholipase A2 group XV [Cricetulus griseus (Chinese hamster)]	ND	ND
**Cathepsin D** ^a^	Cathepsin D OS = Cricetulus griseus OX = 10029 GN = H671_3g9701 PE = 3 SV = 1	ND	ND
**Peroxiredoxin‐1** ^a^	Peroxiredoxin‐1 OS = Cricetulus griseus OX = 10029 GN = I79_002954 PE = 4 SV = 1	ND	ND

Abbreviations: CFB, concentrated fed‐batch; HCPs, host cell proteins.

^a^ High risk HCP.

### 3.5 Risk of HCPs in CFB product was mitigated by applying several control strategies

**FIGURE 6:**
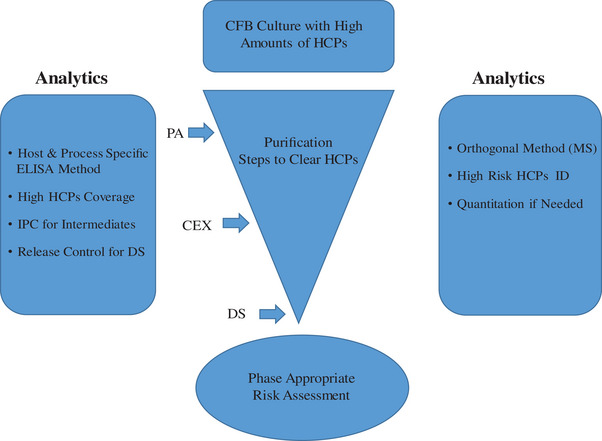
Host cell proteins (HCPs) control strategies for concentrated fed‐batch (CFB) products.

Due to the potential immunogenicity and compromise of product stability, the total HCP residue in the final therapeutic antibody drugs is suggested to be less than 100 ppm. Furthermore, risk control and assessment of HCP impurities in the final therapeutic antibodies are needed, especially for the CFB cultured high density therapeutic antibody products. As shown in Figure 6 and Table 3, risk assessment of HCPs can be divided into three components of severity, occurrence, and detectability. Clinical safety data and product stability are important indicators to evaluate for the severity of HCP. A self‐developed specific ELISA method and novel non‐denaturing enzyme digestion LC‐MS/MS technology were designed to identify low abundance HCPs of different process stages of CFB products.

**TABLE 3:** Risk assessment for the HCPs in CFB product

						Risk priority: Severity^*^ Occurrence^*^ Detectability	Overall risk
Severity		Occurrence		Detectability			
Score	Explanation	Score	Explanation	Score	Explanation		
3	1) No obvious proteases related PS80 degradation observed;	2	1) Purification process shows continuous and robust HCP clearance;	2	1) A self‐developed host cell and process specific ELISA are used as the routine release and IPC method	12	Low
	2) More than 300 persons’ safety data indicating no immunogenicity issue		2) All the batches have very low residual HCPs contents and are within the release specification		2) MS used as the orthogonal method to identify high risk HCPs		

* Multiply of the severity score, occurrence score and detectability score.

Abbreviations: CFB, concentrated fed‐batch; ELISA, enzyme‐linked immunosorbent assay; HCPs, host cell proteins.

## 4 CONCLUDING REMARKS

This work evaluated the combinatorial control strategies for HCPs from high density CFB culture processes. Since the harvest fluids from CFB processes usually have about 10‐fold higher total content of HCPs than traditional FB processes, we developed one host cell and process specific ELISA method and an orthogonal LC‐MS/MS method to assess and mitigate the risk of HCPs in CFB mode products. The host cell and process specific ELISA method was developed through null host strain protein harvest, immunization, polyclonal antibody purification, and specific ELISA kit preparation. The LC‐MS/MS method combined a non‐denaturing enzyme digestion, high separation liquid phase system, high sensitivity & high throughput data dependent acquisition (DDA) on a Thermo/QE‐HF‐X mass spectrometer to deeply comprehend profiles of HCPs. By applying both methods, the HCP clearing trend through multiple purification steps was well monitored. Through the combination of these control strategies, the risk of HCPs in CFB mode products may be largely mitigated.

## AUTHOR CONTRIBUTIONS

Jun Lin designed and led the project, wrote and reviewed the manuscript. Yiling Lu developed the novel LC‐MS/MS method, analyzed the samples by LC‐MS/MS, and wrote the manuscript. Tianze Bian prepared the HCPs pools, responsible for specific HCPs determination kit development, and method optimization. Dan Liu and Mingjun Ma contributed to the LC‐MS/MS analysis of HCPs from null cell harvest and intermediate/drug substance. Zhen Gao and Jiemin Chen prepared the graphs and reviewed the manuscript. Dianwen Ju designed the project and reviewed the manuscript. Xing Wang lead the immunization and reviewed the manuscript.

## CONFLICT OF INTEREST STATEMENT

The authors declare no conflict of interests.

## Data Availability

The data that support the conclusions of this study are available on request from the corresponding author.
